# Evaluation of the differentiation of benign and malignant breast lesions using synthetic relaxometry and the Kaiser score

**DOI:** 10.3389/fonc.2022.964078

**Published:** 2022-10-11

**Authors:** Lingsong Meng, Xin Zhao, Jinxia Guo, Lin Lu, Meiying Cheng, Qingna Xing, Honglei Shang, Kaiyu Wang, Bohao Zhang, Dongmei Lei, Xiaoan Zhang

**Affiliations:** ^1^ Department of Radiology, The Third Affiliated Hospital of Zhengzhou University, Zhengzhou, China; ^2^ Academy of Medical Sciences, Zhengzhou University, Zhengzhou, China; ^3^ General Electric (GE) Healthcare, MR Research China, Beijing, China; ^4^ Henan Key Laboratory of Child Brain Injury, Institute of Neuroscience and the Third Affiliated Hospital of Zhengzhou University, Zhengzhou, China; ^5^ Department of Pathology, The Third Affiliated Hospital of Zhengzhou University, Zhengzhou, China

**Keywords:** Synthetic MRI, Kaiser score, BI-RADS, Breast Imaging Reporting and Data System, breast lesion, malignancy

## Abstract

**Objective:**

To investigate whether there is added value of quantitative parameters from synthetic magnetic resonance imaging (SyMRI) as a complement to the Kaiser score (KS) to differentiate benign and malignant breast lesions.

**Materials and methods:**

In this single-institution study, 122 patients who underwent breast MRI from March 2020 to May 2021 were retrospectively analyzed. SyMRI and dynamic contrast-enhanced MRI were performed using a 3.0-T system. Two experienced radiologists independently assigned the KS and measured the quantitative values of T1 relaxation time (T1), T2 relaxation time (T2), and proton density (PD) from SyMRI. Pathology was regarded as the gold standard. The diagnostic values were compared using the appropriate statistical tests.

**Results:**

There were 122 lesions (86 malignant and 36 benign) in 122 women. The T1 value was identified as the only independent factor for the differentiation of malignant and benign lesions. The diagnostic accuracy of incorporating the T1 into the KS protocol (T1+KS) was 95.1% and 92.1% for all lesions (ALL) and The American College of Radiology (ACR) Breast Imaging Reporting and Data System (BI-RADS) category 4 lesions, respectively, which was significantly higher than that of either T1 (ALL: 82.8%, *P* = 0.0001; BI-RADS 4: 78.9%, *P* = 0.002) or KS (ALL: 90.2%, *P* = 0.031; BI-RADS 4: 84.2%, *P* = 0.031) alone. The sensitivity and specificity of T1+KS were also higher than those of the T1 or KS alone. The combined diagnosis could have avoided another 15.6% biopsies compared with using KS alone.

**Conclusions:**

Incorporating T1 into the KS protocol improved both the sensitivity and specificity to differentiate benign and malignant breast lesions, thus avoiding unnecessary invasive procedures.

## Introduction

Breast cancer is the most frequently diagnosed malignant tumor in women and is currently the second leading cause of female cancer-related death (1, 2). Early detection and accurate characterization can reduce the death rates of patients with breast cancer significantly (3, 4). Dynamic contrast-enhanced magnetic resonance imaging (DCE-MRI) has played a critical role in the detection and characterization of breast lesions (5–7). The American College of Radiology Breast Imaging Reporting and Data System (BI-RADS) lexicon, based on DCE-MRI, provides a standardized and structured description of how breast lesions are enhanced and thus is widely used in current clinical practice (8).

However, the BI-RADS (fifth version) lexicon does not provide a definitive classification scheme, although it emphasizes morphological features and enhancement kinetics. The Kaiser score (KS) is a clinical decision rule that places the independent diagnostic BI-RADS lexicon criteria, including root sign, time-signal intensity curve (TIC) types, lesion margins, internal enhancement patterns, and peritumoral edema, as described in previous studies (9–14), into an intuitive machine-learning flowchart to grade a breast lesion in much more detail (11, 14–17). The KS value ranges from 1 to 11, each of which is associated with a distinct probability of malignancy (10). If the score exceeds 4, a biopsy is needed (15, 16). Although the KS is not widely utilized and has not been comprehensively assessed in comparison to BI-RADS, it was demonstrated to be less subjective in the assessment of breast lesions, which has been validated in suspicious MRI-only lesions (14) and in lesions that present as mammography-related calcifications (16). Methods to improve the differentiation performance using the KS are still under exploration. A multicenter study (13), as well as our recent study (18), investigated a combination of the KS and the apparent diffusion coefficient value; however, it did not benefit the diagnosis of benign and malignant breast lesions.

Synthetic magnetic resonance imaging (SyMRI) with magnetic resonance imaging compilation (MAGiC) is a recently proposed multi-dynamic multi-echo sequence, which can simultaneously generate quantitative T1 relaxation time (T1), T2 relaxation time (T2), and proton density (PD) maps in a clinically acceptable acquisition time (19–21). Several studies have demonstrated the usefulness of the quantitative values from SyMRI in the differential diagnosis of breast cancers (22–25). Meng et al. (22) investigated T1 and T2 values from SyMRI and found their combination performed better in the diagnosis of breast cancer compared with T1 or T2. Matsuda et al. (23) demonstrated an improved diagnostic accuracy of malignant and benign breast masses when combining the T1 from SyMRI and DCE-MRI. Gao et al. (24) incorporated the T2 and PD of SyMRI, and the apparent diffusion coefficient of diffusion-weighted imaging (DWI) to differentiate malignant and benign breast lesions and showed improved specificity in comparison with BI-RADS.

Therefore, the present study aimed to determine whether the quantitative parameters of the SyMRI could be helpful when incorporated with the KS to differentiate benign and malignant breast lesions and their potential to avoid unnecessary biopsies.

## Materials and methods

### Patient population

**Figure 1:**
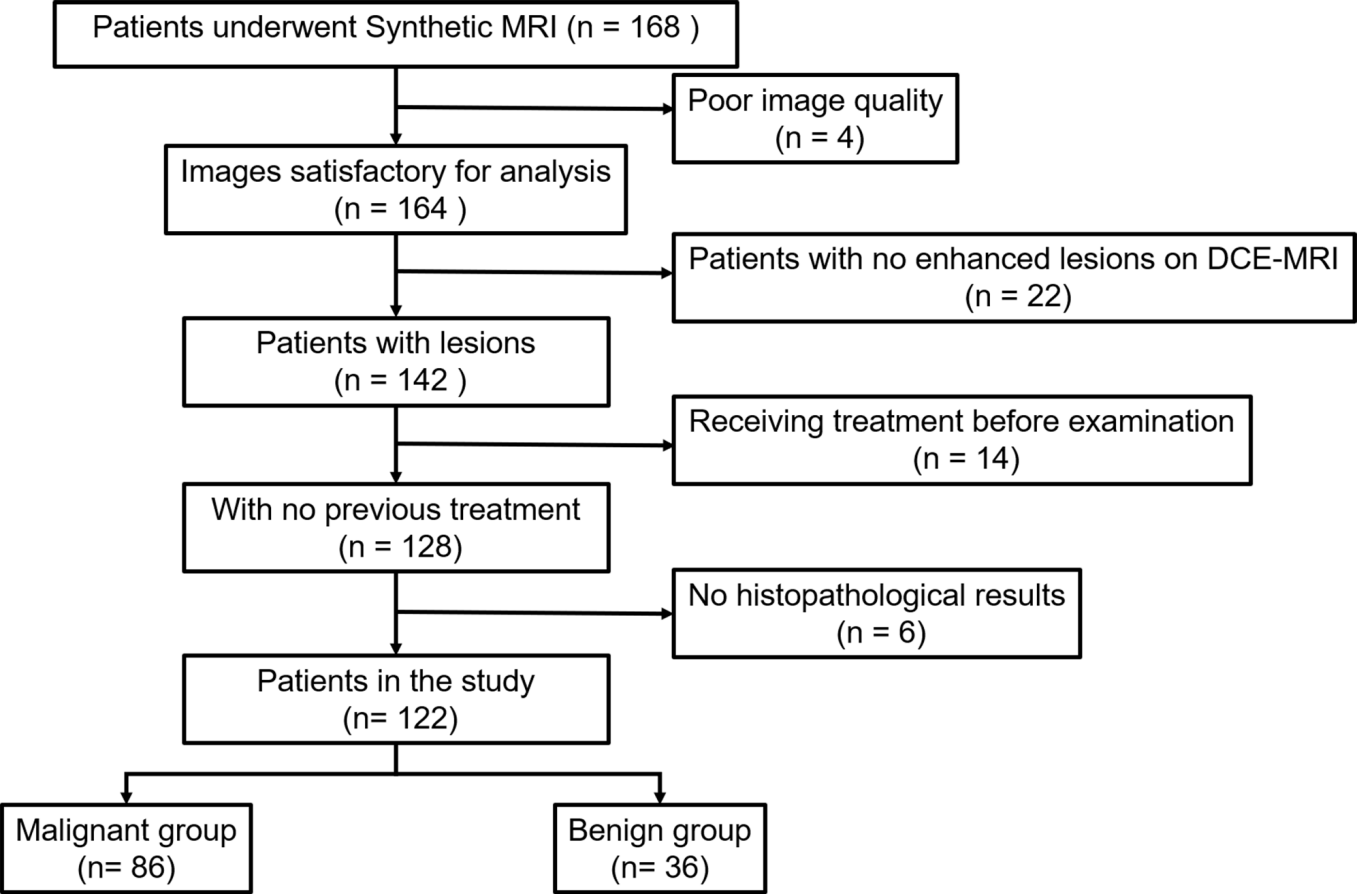
Flowchart of patient selection. *DCE-MRI*, dynamic contrast-enhancement magnetic resonance imaging.

This retrospective study was approved by our Institutional Review Board and informed consent was obtained from all patients. From March 2020 to May 2021, we consecutively reviewed 168 female patients with suspicious findings (rated as BI RADS 0, 4, and 5 after mammography or breast ultrasonography) who underwent MRI examinations at our institution. A total of 46 patients were excluded for the following reasons: (1) Patients with no enhanced lesions on DCE-MRI (n = 22); (2) patients who underwent biopsy or chemotherapy before MR examination (n = 14); (3) patients without available histopathological results (n = 6); and (4) imaging with artifacts at synthetic MRI (n = 4). Ultimately, 122 patients were included in this study (**Figure 1**). The MRI BI-RADS categories of the lesions were extracted from our picture archiving and communication system. Lesion size was defined as the maximal diameter of the lesion (cm) on the peak enhanced phase (according to the time-intensity curve) of the DCE-MRI. Lesions smaller than 0.8 cm were not included in this study due to the possible bias resulting from the partial volume effect. When multiple breast lesions were present, the largest lesion was analyzed. The histopathological results of all breast lesions were diagnosed by biopsy or pathology after surgery.

### MRI technique

All image data were acquired using a 3-T MRI system (SIGNA Pioneer, GE Healthcare, Waukesha, WI, USA) with an 8-channel breast coil. All patients were examined in the prone position. The scan protocols included axial T1 weighted imaging (T1WI), T2 weighted imaging (T2WI) with fat saturation, and SyMRI with MAGiC followed by DCE-MRI. For SyMRI with MAGiC (scan time: 5:12 min), the data were acquired using a commercially available multi-dynamic multi-echo sequence with two echo times and four saturation delay times. Then, the real and imaginary images of SyMRI were imported to SyMRI 11.0 software (Synthetic MR, Linköping, Sweden) to generate quantitative parametric maps (T1, T2, and PD maps) for further analysis. The settings of all MR acquisitions are summarized in **Supplementary materials Table S1**.

### Interpretation of Kaiser score based on DCE MRI

The KS incorporates the five most common diagnostic features of the BI-RADS: root sign, TIC types, lesion margins, internal enhancement patterns, and peritumoral edema, and was determined according to previous studies (10, 11, 13, 14). The TIC was generated by drawing the region of interest (ROI) with the largest increase of the early signal on DCE images (26). Two experienced breast radiologists (M.Y.C. and L.L. with 10 and 15 years of experience in reading breast MR images, respectively) were required to interpret all examinations following the KS system, as reported in the literature (10). Both readers were blinded to the histological results and BI-RADS categories. Disagreements regarding the KS categories were resolved by consensus. In this study, lesions with a KS greater than 4 were diagnosed as malignant, otherwise, the lesions were diagnosed as benign (15).

### Interpretation of quantitative maps of SyMRI

All qualitative maps (T1, T2, and PD maps) were independently analyzed by two radiologists (L.S.M. and M.Y.C. with 5 and 10 years of experience in reading breast MR images, respectively) using the open-source software ITK-SNAP (http://www.itksnap.org/pmwiki/pmwiki.php, version 3.8). Both readers were blinded to the final histopathological results and the KS categories. The ROIs were drawn manually to encompass the areas of the lesions in the maximum image section using the DCE-MRI as the reference. The ROI included the largest solid areas of the lesion, while the areas with visible necrosis, cystic change, or hemorrhage were excluded (24). The qualitative parameters including the T1, T2, and PD values in the ROIs were calculated, as well as T1/T2.

### Statistical analysis

The intraclass correlation coefficients were calculated to test the inter-reader agreement of the KS and quantitative parameters from SyMRI. After the normality and homogeneity of variances were examined, the quantitative SyMRI parameters (T1, T2, PD, and T1/T2) and the KS in malignant and benign lesions were compared using the independent sample *t*-test or Mann-Whitney *U*-test. Then, binomial logistic regression analysis was applied to the quantitative SyMRI parameters with significant differences to explore the independent factors based on stepwise selection and Akaike information criterion. Receiver operating characteristic (ROC) analysis was used to evaluate the performance of the independent quantitative factors from SyMRI to differentiate malignant from benign lesions. The optimal thresholds for differentiating malignant from benign lesions were obtained at the largest Youden index (Youden index = sensitivity + specificity-1). The sensitivity, specificity, positive predictive value (PPV), negative predictive value (NPV), and accuracy, were compared between the KS and the combination of KS and the independent quantitative factors. The McNemar test was used in the comparisons to assess the potential of avoiding unnecessary biopsies for each diagnostic strategy.

All data were analyzed using the statistical software SPSS 26.0 (IBM Corp, Armonk, NY, USA) and MedCalc 19.8 (MedCalc Software Ltd., Ostend, Belgium). *P* < 0.05 was considered statistically significant.

## Results

### Study population

A total of 122 patients (mean age, 46.6 ± 11.6 years; age range, 18–68 years) with 122 lesions (86 malignant (mean age 50.3 ± 9.9 years and age range 21–68 years); 36 benign (mean age 38.0 ± 10.7 years and age range 18–58 years)) were included in the study. Among the malignant lesions,77 lesions were invasive ductal carcinoma, 6 were ductal carcinoma *in situ*, 2 were invasive lobular carcinoma, and 1 was mucinous carcinoma. Among the benign lesions, 26 lesions were fibroadenoma, 4 were papilloma, 3 were inflammation, and 3 were adenosis. The mean lesion diameter was 2.9 ± 1.7 cm for malignant lesions and 2.4 ± 2.0 cm for benign lesions.

### Summary of KS findings

The intraclass correlation coefficient for KS was 0.969 (95% confidence interval: 0.956–0.978), indicating perfect agreement between the two readers. The results of KS based on DCE-MRI are summarized in **Table 1**. In our study, the KS of malignant lesions was significantly higher than that of benign lesions (*P* < 0.001). The KS cutoff value was 4 and the diagnostic accuracy of the application of KS was 90.2% (110/122).

**Table 1:** Statistics of the lesions’ Kaiser scores.

Characteristic	Malignant (n = 86)	Benign (n = 36)
Lesion type on DCE-MRI		
Mass enhancement (n = 99)	67 (77.9%)	32 (88.9%)
Non-mass enhancement (n = 23)	19 (22.1%)	4 (11.1%)
KS value^a^	9 (7–10)	2 (1–4.75)
KS category		
Positive (KS > 4) (n = 92)	83 (96.5%)	9 (25%)
Negative (KS ≤ 4) (n = 30)	3 (3.5%)	27 (75%)
BI-RADS		
3 (n = 4) 4 (n = 76) 5 (n = 42)	0 (0%) 44 (51.2%) 42 (48.8%)	4 (11.1%) 32 (88.9%) 0 (0%)

a Data are medians and interquartile ranges. DCE-MRI, dynamic enhanced magnetic resonance; KS, Kaiser score; BI-RADS, breast imaging reporting and data system.

### Summary of quantitative T1/T2/PD analysis

**Figure 2:**
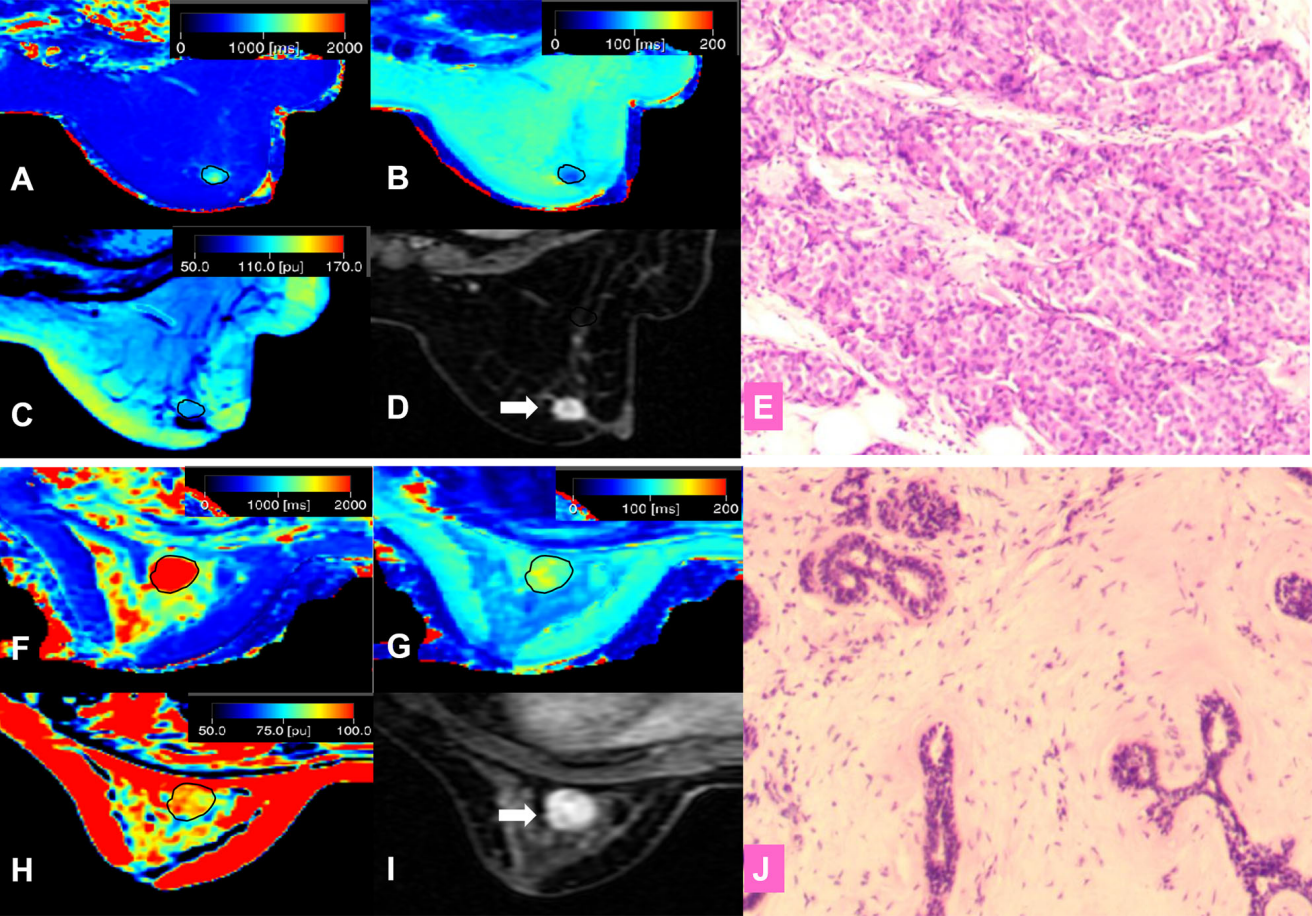
**(A–E)** A 57-year-old female patient with invasive ductal carcinoma in the right breast **(D,** white arrow**)**. Photomicrograph **(E)** showing higher cellular density and decreased extracellular space. Kaiser score = 9; T1 relaxation time = 1192.80 ms; T2 relaxation time = 89.02 ms; PD = 98.36 pu. **(A)** T1 map; **(B)** T2 map; **(C)** PD map; **(D)** Contrast-enhanced MRI; **(E)** Pathological image (hematoxylin and eosin (H & E), 100×). **(F–J)** A 42-year-old female patient with a fibroadenoma in the left breast (**I**, white arrow). Kaiser score = 2; T1 relaxation time = 2276.46 ms; T2 relaxation time = 129.32 ms; PD = 95.16 pu. Photomicrograph **(J)** showing hypercellular stroma. **(F)** T1 map; **(G)** T2 map; **(H)** PD map; **(I)** Contrast-enhanced MRI; **(J)** Pathological image (H & E, 100×).

The intraclass correlation coefficients calculated by two radiologists for T1, T2, and PD were all greater than 0.92, which indicated excellent agreement. The detailed results of the agreement analysis are summarized in **Supplementary materials Table S2**. Upon univariate analysis, significantly lower values were found for malignant than for benign lesions for T1 (1398.97 ± 393.14 ms *vs*. (1968.88 ± 467.49 ms), T2 (85.95 ± 24.24 ms *vs*. 108.46 ± 29.49 ms), and T1/T2 (16.48 ± 3.47 *vs*. 18.67 ± 4.02), while there was no significant difference for PD (**Table 2**). Multivariate analysis demonstrated that T1 was the independent prediction factor for the risk of lesion malignant (*P* = 0.016) (**Table 2**). Clinical examples are provided in **Figure 2**.

**Table 2:** Comparison of quantitative T1/T2/PD Measures between Benign and Malignant Groups.

Parameters	Malignant (n = 86)	Benign (n = 36)	Univariate analysis	Multivariate analysis		
			*P* ^b^	OR	95% CI	*P* ^c^
T1 (ms)^a^	1398.97 ± 393.14	1968.88 ± 467.49	<0.001*	0.9926	0.9866–0.9986	0.016*
T2 (ms)^a^	85.95 ± 24.24	108.46 ± 29.49	<0.001*	1.0591	0.9812–1.1431	0.141
PD (pu)^a^	84.76 ± 18.95	86.25 ± 14.66	0.271			
T1/T2^a^	16.48 ± 3.47	18.67 ± 4.02	0.004*	1.5003	0.8849–2.5437	0.132

a Data are the mean ± standard deviation.

^*^P values < 0.05 were considered statistically significant.

b P values calculated by the Mann-Whitney U test.

c P values calculated by the multivariate logistic regression analysis for each variable.

T1, longitudinal relaxation time; T2, transverse relaxation time; PD, proton density; T1/T2, ratio of T1 and T2; OR, odds ratio; CI, confidence interval.

### Diagnostic performance of T1, KS, and their combination

The diagnostic performances of T1 and the KS are summarized in **Table 3**. On the one hand, when assessing all breast lesions, the KS showed a satisfactory sensitivity (96.5%) but slightly low specificity (75%). On the other hand, the sensitivity of T1 (83.7%) was significantly lower than that of the KS (*P* = 0.003), but the specificity (80.6%) was slightly higher than that of the KS (*P* = 0.727). The diagnostic accuracy, PPV, and NPV of the KS were 90.2%, 90.0%, and 90.2%, respectively. In the assessment of MR BI-RADS category 4 lesions, the KS also showed a higher sensitivity of 93.2% (*P* = 0.07), but a slightly lower specificity of 71.9% (*P* = 0.727), compared with those of T1.

**Table 3:** Diagnostic performance for different parameters or their combination in differentiating malignant and benign breast lesions.

Lesion type	Parameters	Cutoff	Sensitivity (%)	Specificity (%)	PPV (%)	NPV (%)	Accuracy (%)	AUC	*P* ^a^	*P* ^b^	*P* ^c^
All lesions	T1 (ms)	1595.94	83.7 (72/86)	80.6 (29/36)	91.1 (72/79)	67.4 (29/43)	82.8 (101/122)	0.875	0.003*†	0.727†	0.078†
	KS	/	96.5 (83/86)	75.0 (27/36)	90.2 (83/92)	90.0 (27/30)	90.2 (110/122)	0.914	1.000‡	0.063‡	0.031*‡
	T1 + KS	/	97.7 (84/86)	88.9 (32/36)	94.4 (84/89)	95.5 (32/33)	95.1 (116/122)	0.933	0.001*‖	0.250‖	<0.001*‖
BI-RADS 4 lesions											
	T1 (ms)	1595.94	79.5 (35/44)	78.1 (25/32)	83.3 (35/42)	73.5 (25/34)	78.9 (60/76)	0.841	0.070†	0.727†	0.455†
	KS	/	93.2 (41/44)	71.9 (23/32)	82.0 (41/50)	88.5 (23/26)	84.2 (64/76)	0.863	1.000‡	0.063‡	0.031‡
	T1 + KS	/	95.5 (42/44)	87.5 (28/32)	91.3 (42/46)	93.3 (28/30)	92.1 (70/76)	0.915	0.016*‖	0.250‖	0.002‖

P^a^, P^b^, and P^c^ values were calculated using the McNemar test to compare the differences in sensitivity, specificity, and accuracy, respectively.

†P values (KS vs. T1); ‡P values (KS vs. T1+KS); ‖P values (T1 vs. T1+KS).

^*^P values < 0.05 were considered statistically significant.

PPV, positive predictive value; NPV, negative predictive value; AUC, area under the curve; T1, longitudinal relaxation time; KS, Kaiser score; BI-RADS, breast imaging reporting and data system.

**Figure 3:**
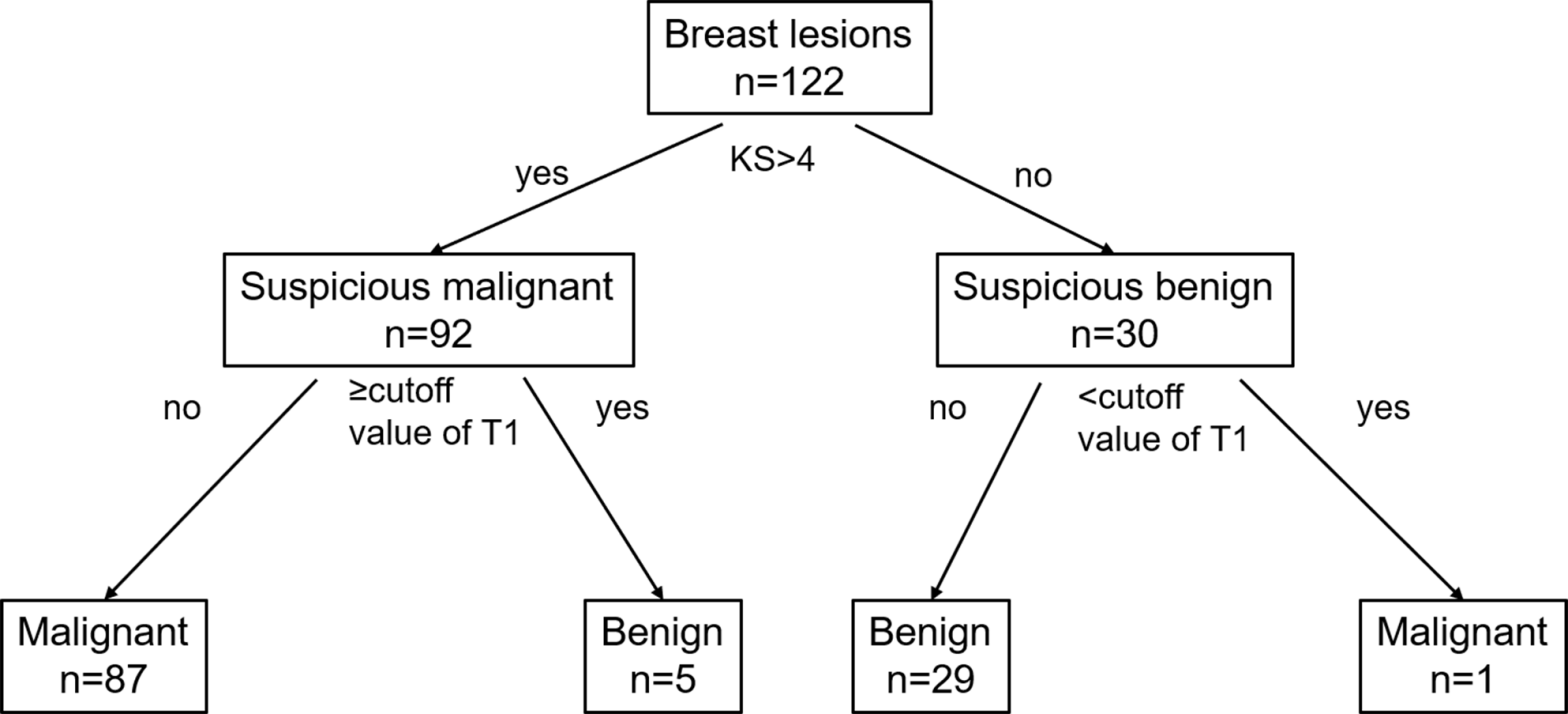
Flowchart for the combination of the KS and SyMRI in the differential diagnosis of breast lesions. *KS*, Kaiser score; *T1*, longitudinal relaxation time.

T1 was the only independent factor; therefore, instead of incorporating the T1 and KS in a regression model directly, we established a diagnostic protocol with KS and T1, as shown in **Figure 3**, which was designed to take advantage of KS’s sensitivity and T1’s specificity. The lesion was categorized first using the KS. If the KS result was the same as the T1 differentiation based on the optimal threshold from the ROC analysis, the diagnosis was kept, otherwise, it would be changed.

**Figure 4:**
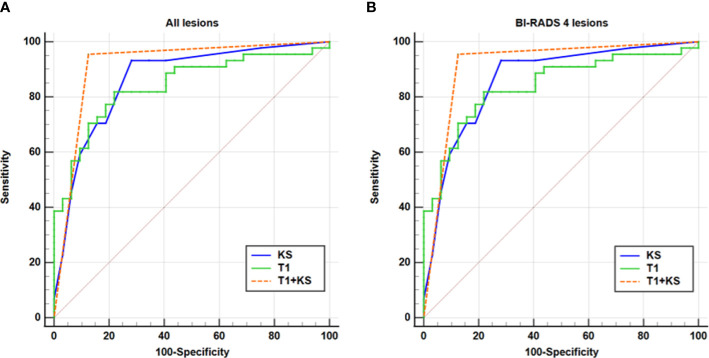
ROC analysis for T1, KS, and T1+KS. The ROC curves illustrate that a higher diagnostic value (i.e., higher sensitivity, specificity, and larger AUC) was reached for the combination of T1 and KS (T1+KS) in assessing all lesions **(A)** and BI-RADS 4 lesions **(B)**, compared with that using T1 and KS alone.

In comparison with T1 or KS alone, the combination of the T1 and KS acquired significantly higher accuracy in the diagnosis of all breast lesions (*P* < 0.05) and BI-RADS 4 lesions (*P* < 0.05). The combined method also showed higher area under the curve (AUC), sensitivity, specificity, PPV, and NPV when assessing all lesions and category 4 lesions (**Table 3**, **Figure 4**).

### Potential for avoiding unnecessary biopsies

In this study, 55% (44/76) of the BI-RADS 4 lesions were pathologically proven to be malignant. The use of T1 led to nine false-negative diagnoses, including three ductal carcinomas *in situ*, five invasive ductal carcinomas, and one mucinous carcinoma. Meanwhile, utilizing the KS missed one ductal carcinoma *in situ*, one invasive ductal carcinoma, and one mucinous carcinoma. When applying the combination of T1 and KS, the false-negative lesions were one ductal carcinoma *in situ* and one mucinous carcinoma.

**Figure 5:**
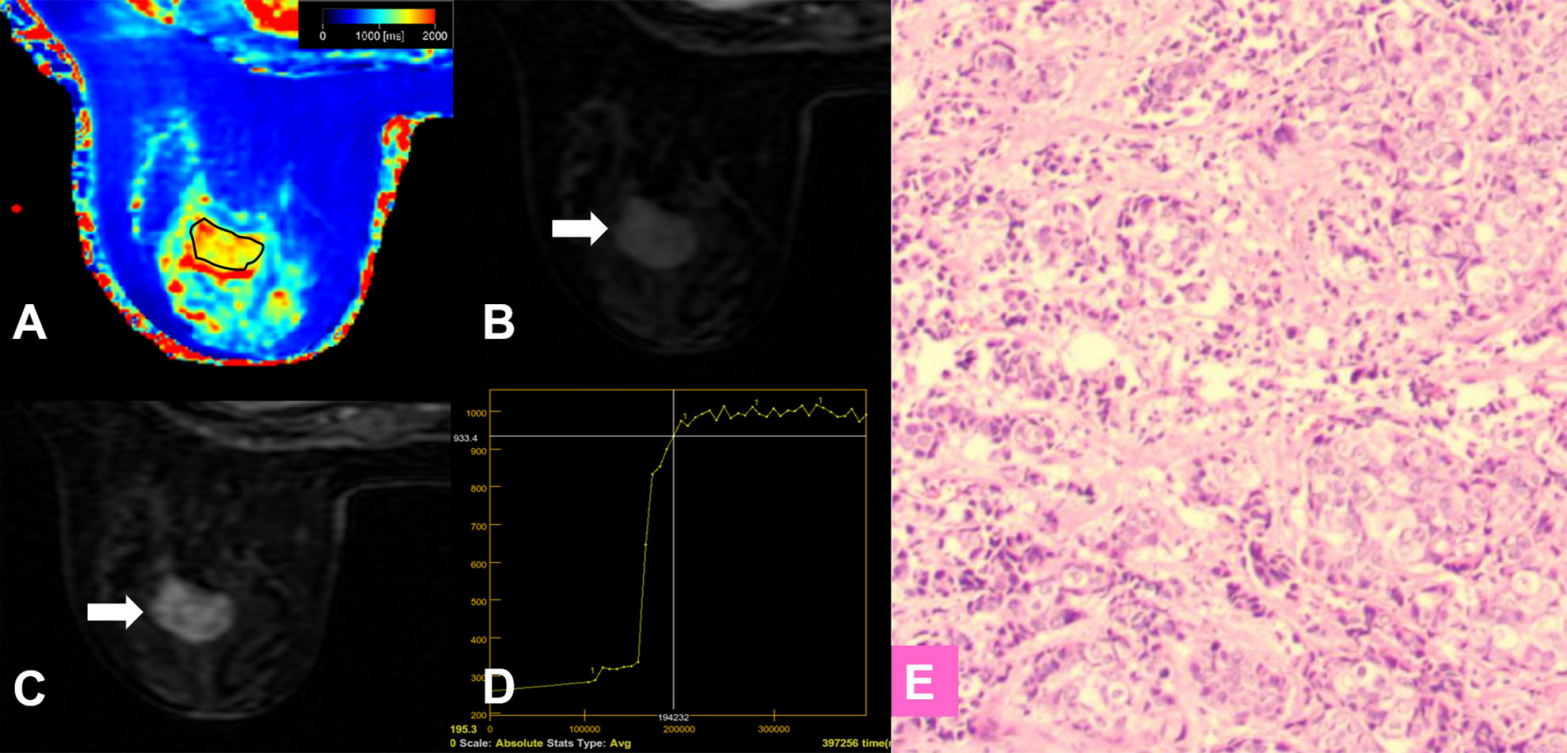
False-negative lesion identified by the KS. **(A–E)** A 56-year-old female patient: MRI showed a mass lesion in the left breast [**(B, C)**, white arrow]. The lesion showed no root sign, circumscribed margins **(B, C)**, and a plateau enhancement curve type **(D)**. **(A)** T1 map, **(B)** Early contrast-enhanced MRI, **(C)** Delayed contrast-enhanced MRI, **(D)** TIC curve, **(E)** Pathological image (H&E, 100×). The Kaiser score was 2 and the lesion was classified as negative for malignancy. The T1 relaxation time was 1449.45 ms, which was lower than the cutoff value of 1595.94 ms. The combined diagnosis using the KS and T1 value identified malignancy. Histopathology revealed an invasive ductal carcinoma **(E)**.

**Figure 6:**
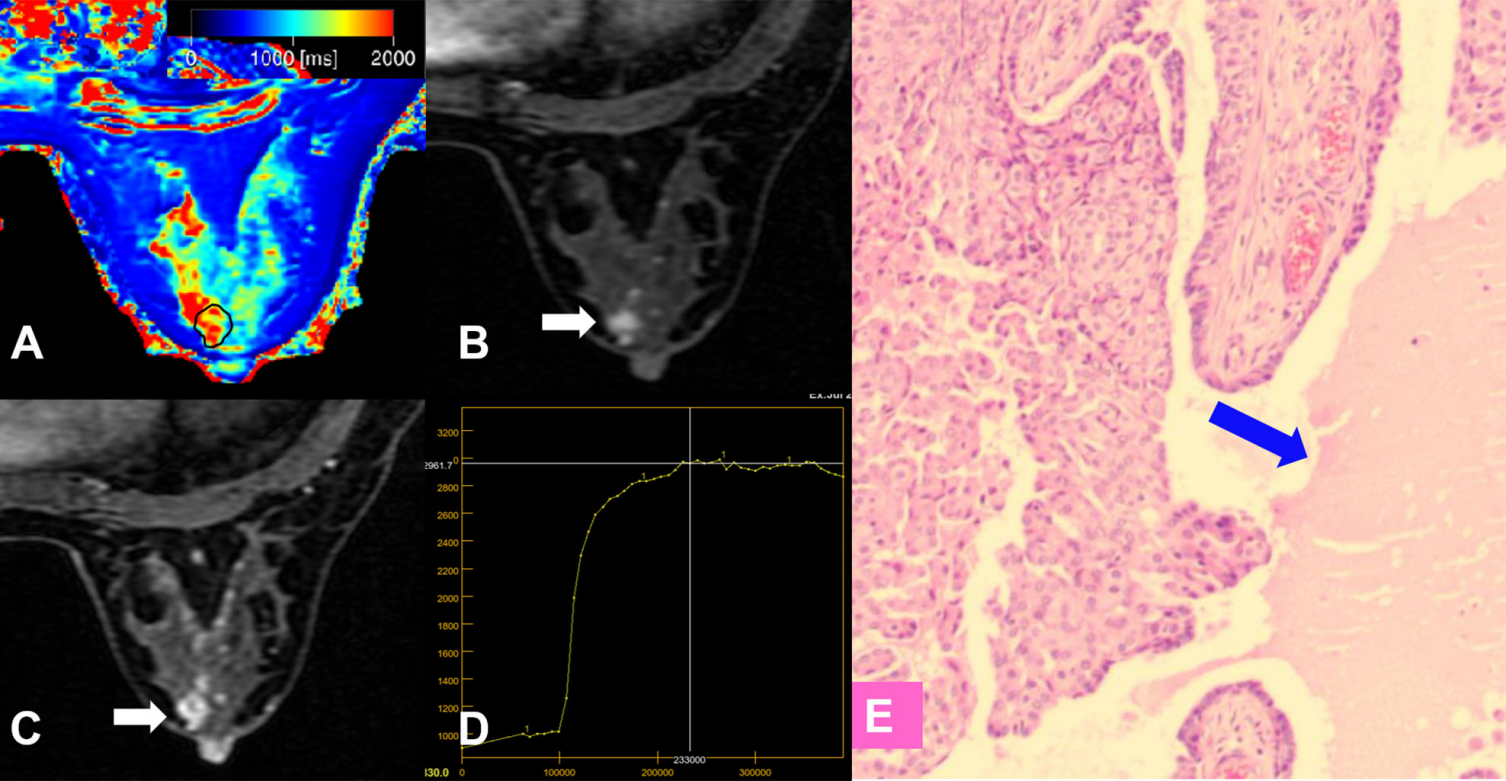
False-positive lesion identified by the KS. **(A–E)** A 45-year-old female patient: MRI showed a mass lesion in the right breast [**(B, C)**, white arrow]. The lesion showed no root sign, irregular margins **(B, C)**, and a plateau enhancement curve type **(D)**. **(A)** T1 map, **(B)** Early contrast-enhanced MRI, **(C)** Delayed contrast-enhanced MRI, **(D)** TIC curve, **(E)** Pathological image (H&E, 100×): an area of edema was seen within the lesion (blue arrow). The Kaiser score was 5 and was classified as positive for malignancy. The T1 relaxation time was 1711.24 ms, which was higher than the cutoff value of 1595.94 ms. The combined diagnosis using the KS and T1 value identified a benign lesion. Histopathology revealed a papilloma **(E)**.

**Figure 7:**
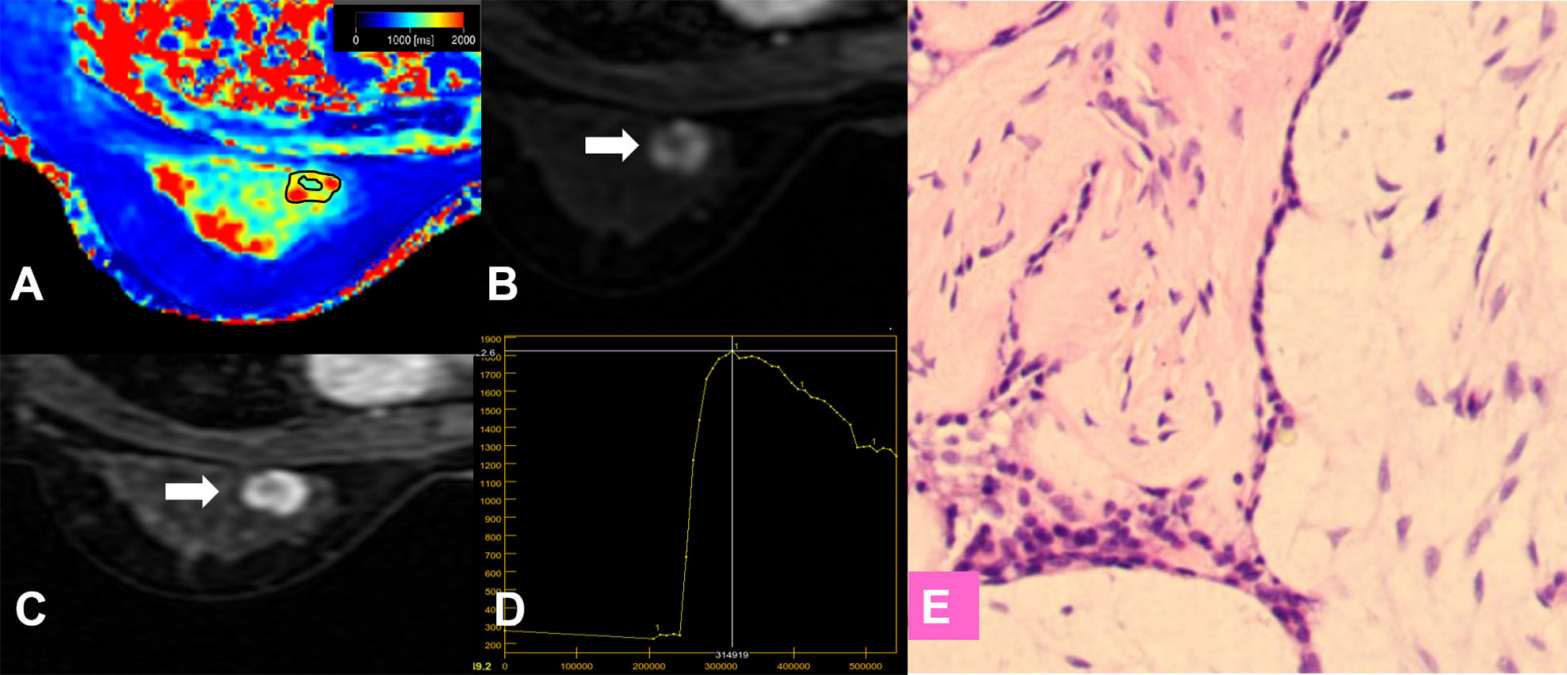
False-positive lesion identified by the KS. **(A–E)** A 27-year-old female patient: MRI showed a mass lesion in the left breast [**(B, C)**, white arrow]. The lesion showed no root sign, inhomogeneous enhancement **(B, C)**, and a washout enhancement curve type **(D)**. **(A)** T1 map, **(B)** Early contrast-enhanced MRI, **(C)** Delayed contrast-enhanced MRI, **(D)** TIC curve, **(E)** Pathological image (H&E, 100×). The Kaiser score was 8 and was classified as positive for malignancy. The T1 relaxation time was 1727.16 ms, which was higher than the cutoff value of 1595.94 ms. The combined diagnosis using the KS and T1 value identified a benign lesion. Histopathology revealed a fibroadenoma **(E)**.

The use of the KS could have avoided 71.9% (23/32) of unnecessary biopsies. This number increased by 15.6% when the combination of T1 and KS was used. The details of the false-negative and false-positive lesions are summarized in **Table 4**. Clinical examples are provided in **Figures 5**–**7**.

**Table 4:** Detailed information of the false-negative and false-positive lesions diagnosed by T1, KS, and their combination.

	False negatives	n	False positives	n
T1	Ductal carcinoma *in situ*	3	Fibroadenoma	3
	Invasive ductal carcinoma	5	Papilloma	2
	Mucinous carcinoma	1	Inflammation	2
KS	Ductal carcinoma *in situ*	1	Fibroadenoma	5
	Invasive ductal carcinoma	1	Papilloma	2
	Mucinous carcinoma	1	Inflammation	2
T1+KS	Ductal carcinoma *in situ*	1	Fibroadenoma	2
	Mucinous carcinoma	1	Papilloma	1
			Inflammation	1

KS, Kaiser score; T1, longitudinal relaxation time.

## Discussion

In this study, the quantitative parameters from SyMRI, KS, and their combination were assessed in the differentiation of malignant and benign breast lesions. Multivariate analysis showed that the T1 was the only significant quantitative parameter derived from SyMRI that could independently distinguish malignant from benign breast lesions. Combining T1 and the KS indicated much better performance in comparison with each single parameter, allowing the differentiation both all lesions and those lesions initially assigned to BI-RADS category 4.

The category 3 lesions were pathologically proven to be benign, and the category 5 lesions were malignant. The misdiagnosed cases were mainly distributed in category 4 lesions. Our results showed that the KS resulted in high sensitivity and specificity, in line with the previous studies (13, 18). In this study, the accuracy of the KS (cutoff value > 4) in the differential diagnosis of categories 3 and 5 was 100%, while there were 3 false negatives and 9 false positives when assessing the category 4 lesions. A simple machine-learning strategy is used in the KS *via* a decision tree (10, 12, 14), which takes full advantage of the independent diagnostic BI-RADS lexicon criteria and uses each of them as a meaningful node to help the diagnosis decision.

With various tissue components and microstructural alterations, such as the tissue water and fat content, macromolecule concentration, and hydration state (27, 28) in malignant or benign lesions, the T1 and T2 values might change in a different way or degree. In this study, we found that both the T1 and T2 values of malignant lesions were significantly lower than those of the benign ones, which was in line with previous studies (29, 30). The histopathological image of the benign lesions had the characteristics of a lower cellular density and hypercellular stroma. In contrast, the high cellular density, small extracellular space, and decreased free water content in malignant lesions, resulting from their vigorous proliferation, might be the main reasons for larger T1 and T2, as explained previously (30, 31).

For T1 quantification, there are discrepant results in existing investigations show discrepant results. We found that the mean T1 values of benign lesions were significantly higher than those of malignant ones. Some studies (22, 23) presented conflicting synthetic T1 values in benign and malignant lesions. The possible reasons for this result were as follows: The ROI generating methods might have an effect on the location of the lesion area and the quantification values (32, 33). Matsuda et al. (23) drew the ROI on synthetic T2W images while Meng et al. (22) and our study obtained the ROI from the DCE images. Another reason is the type of enrolled benign lesion. Our study included a relatively high number of benign breast lesions, and fibroadenoma accounted for the highest proportion (72.2%). Fibroadenomas are the most common benign breast lesions with characteristics of a lower cellular density and hypercellular stroma that indicates a high free water content (resulting in a high T1 relaxation time). Additionally, myxoid degeneration in fibroadenomas (22) also lengthens the longitudinal relaxation time of T1.

T1 was proven to be the independent predictive factor for the risk of a lesion being malignant and was thus added to joint diagnosis with the KS. Logistic regression was not used for the multi-index combined diagnosis because of its inconvenience in clinical practice. Instead, we added T1 to the KS decision tree, in which T1 was used as a quantitative reference for correction of KS-based diagnosis (**Figure 3**), which was inspired by the study of Dietzel (13).

The combined T1 and KS protocol demonstrated improved performance in differentiating BI-RADS category 4 lesions, which are suspicious of malignancy and are usually recommended for biopsies (34). The probability of malignancy in category 4 varies from 2% to 95% (7, 35), which means a large number of benign lesions would receive unnecessary invasive procedures. which means that a large number of women with benign lesions would receive unnecessary invasive procedures. In our study, 45% (32/76) of the BI-RADS 4 lesions were pathologically proven as benign. The use of the KS could have avoided 71.9% (23/32) of the unnecessary biopsies. This number increased by 15.6% when the combination of T1 and KS was used. The DCE-MRI features of benign and malignant lesions overlap; therefore, the use of the KS might lead to a false-positive diagnosis. The combined method counterbalanced the lack of specificity of the KS and the lack of sensitivity of the T1 value.

The threshold of the quantitative T1 value determined in our study was 1595.94 ms, which was higher compared with the 1.5T system results (1049 ms) reported by Merchant et al. (29). Previous studies have proven that the T1 relaxation time of tissue increases with the field strength (36, 37). However, because of the long acquisition time (38), there are few studies in the 3T system with traditional T1 mapping technology about the T1 relaxation time, nor is there a commonly accepted threshold to assess breast lesions. The number of breast lesions in recent synthetic MRI studies, including this study, was small and the enrolled pathological types were different, which might have affected the quantification of T1 relaxation time. Therefore, further research with a larger sample size and various pathologies is necessary to achieve a more reasonable T1 cutoff value.

Our study has several other limitations. (1) Compared with the apparent diffusion coefficient, the T1 relaxation time, in theory, is the intrinsic parameter related to cell microstructure and composition (36, 37), which might be less affected by the data acquisition system and scanning parameters; however, multi-center studies are still needed to further verify the results. (2) Our study collected a higher number of malignant lesions than benign ones, which might have led to statistical bias, although we applied statistical methods based on the data distribution characteristics carefully. This was because most of the patients had experienced the initial breast screening imaging before, and they received an MRI examination because of symptomatic breast tumors or for the second-look examinations in our institution, a women’s and children’s hospital. A further study recruiting subjects from a screening population might be necessary to confirm the findings of this study in future. (3) The lesions in this study were mostly invasive ductal carcinoma and fibroadenoma. More examples of other types of breast lesions need to be considered and the quantitative relaxation values between different types of benign lesions will also be analyzed in the future. (4) Further combined protocols of KS and the T1 are worthy of exploration to further optimize performance.

## Conclusion

Incorporating T1 into the KS protocol improved both the sensitivity and specificity to differentiate benign and malignant breast lesions, which might help avoid unnecessary invasive procedures.

## Data availability statement

The datasets presented in this article are not readily available because of institutional restrictions. Requests to access the datasets should be directed to Xiaoan Zhang, zxa@zzu.edu.cn.

## Ethics statement

The studies involving human participants were reviewed and approved by the Institutional Review Board of the Third Affiliated Hospital of Zhengzhou University. The patients/participants provided their written informed consent to participate in this study. Written informed consent was obtained from the individual(s) for the publication of any potentially identifiable images or data included in this article.

## Author contributions

Conception and design: LM. Administrative support: XaZ, XZ, and LL. Provision of study materials or patients: MC, DL, and BZ. Collection and assembly of data: LM, HS, and QX. Data analysis and interpretation: LM and JG. Writing - review & editing: LM, JG, and KW. Final approval of manuscript: All authors. All authors contributed to the article and approved the submitted version.

## Funding

This research was funded by The National Natural Science Foundation of China. Grant No. 81870983. At the same time, it was also funded by The Department of science and technology of Henan Province (Grant No. 212102310694) and Major projects of collaborative innovation in Zhengzhou (Grant NO. 18XTZX12009).

## Acknowledgments

We are grateful to the colleagues in the Pathology Department for providing pathological materials.

## Conflict of interest

JG and KW were employed by General Electric (GE) Healthcare.

The remaining authors declare that the research was conducted in the absence of any commercial or financial relationships that could be construed as a potential conflict of interest.

## Publisher’s note

All claims expressed in this article are solely those of the authors and do not necessarily represent those of their affiliated organizations, or those of the publisher, the editors and the reviewers. Any product that may be evaluated in this article, or claim that may be made by its manufacturer, is not guaranteed or endorsed by the publisher.
