# Supplementary material for: Evaluation of the differentiation of benign and malignant breast lesions using synthetic relaxometry and the Kaiser score
Source: Front Oncol. 2022 Oct 11;12:964078. doi: 10.3389/fonc.2022.964078 (PMC9595598; doi:10.3389/fonc.2022.964078)
Supplement: Supplementary file 1 [file Table_1.docx]

**Table S1.** MRI Protocol Parameters

| **Parameters** | **T1WI** | **T2WI** | **SyMRI** | **DCE** |
| --- | --- | --- | --- | --- |
| Scan plane | Axial | Axial | Axial | Axial |
| Sequence | FSE | FSE | MAGiC | SPGR |
| TR (msec) | 763 | 7281 | 7079 | 4.7 |
| TE (msec) | 7 | 77 | 21.4/106.8 | 1.7 |
| Fat suppression | OFF | ON | OFF | ON |
| FOV (cm^2^) | 36 × 36 | 36 × 36 | 36 × 36 | 36 × 36 |
| Matrix | 224 × 352 | 512 × 512 | 320 × 224 | 360 × 360 |
| Slice thickness (mm) | 5 | 5 | 5 | 1.2 |
| Spacing, (mm) | 0 | 0 | 0 | 0 |
| Number of slices | 32 | 32 | 32 | 134 |
| Bandwidth (kHz) | 62.5 | 62.5 | 25 | 142 |
| NEX | 1 | 1 | 1 | 1 |
| Acquisition time (min) | 0:37 | 3:03 | 5:12 | 4:42 |
| Contrast agent (only DCE) | Omni-Scan (GE Healthcare, Shanghai, China), 0.1 mmol/kg body weight | | | |

*TR*, repetition time; *TE*, echo time; *NEX*, number of excitations; *FOV*, field of view; *T1WI*, T1 weighted imaging; *T2WI*, T2 weighted imaging; *SyMRI*, synthetic magnetic resonance imaging; *DCE*, dynamic contrast-enhanced; *FSE*, fast spin echo; *MAGiC*, magnetic resonance imaging compilation

**Table S2.** Inter-reader agreement for each parameter of breast lesions

| **Parameters** | **ICC** | **95% CI** |
| --- | --- | --- |
| T1 | 0.9727 | 0.9612-0.9808 |
| T2 | 0.9205 | 0.8882-0.9438 |
| PD | 0.9802 | 0.9717-0.9861 |
| KS | 0.9693 | 0.9563-0.9784 |

*T1*, longitudinal relaxation time; *T2*, transverse relaxation time; *PD*, proton density; *KS*, Kaiser score; *ICC*, intraclass correlation coefficient; *CI*, confidence interval
